# Viral load suppression among HIV-positive adult patients on the first-line antiretroviral treatment regimen in health facilities in the City of Ekurhuleni, Gauteng province, South Africa

**DOI:** 10.11604/pamj.2024.49.102.42645

**Published:** 2024-12-02

**Authors:** Kwetepe Gladys Mafolo, Lindiwe Cele, Mmampedi Mathibe

**Affiliations:** 1 Department of Public Health, School of Health Care Sciences, Sefako Makgatho Health Sciences University, Ga-Rankuwa, South Africa

**Keywords:** Antiretroviral therapy, clinical characteristics, HIV, record review, viral load, South Africa

## Abstract

**Introduction:**

the Human Immunodeficiency Virus (HIV) remains a global health concern, with South Africa among the hardest hit countries. Viral suppression in HIV patients is a positive treatment outcome when monitoring progress and using antiretrovirals (ART). Despite the Joint United Nations Programme on HIV/AIDS (UNAIDS) 95-95-95 strategy which focuses on monitoring viral suppression, unsuppressed viral loads keep persisting because of several factors. The study sought to determine viral load suppression and contributing factors among adult patients on first-line ART in Ekurhuleni District, South Africa.

**Methods:**

the study reviewed 379 patient files of adult patients on first-line ART between April and June 2018. Standardized tools were used to collect sociodemographic and clinical data. Descriptive data was analyzed using frequency tabulations and percentages, and multivariable logistic regression was used to identify factors associated with viral load suppression.

**Results:**

the study included 379 patient records. Mean age; DS of 38.9 years with standard deviation at 10.1. The prevalence of viral load suppression at 12 months was 75.2% (n=285). Female participants had higher suppression rates 77.7% (n=188) than males 70.8% (n=97), received pre-ART counseling 73.1% (n=277) as well as no history of tuberculosis 92.4% (n=350). Being female with (aOR=7.29, 95% CI: 1.02 - 52.26; p=0.04) and having completed a six-month viral load assessment (aOR=1.85, 95% CI: 1.00 - 3.41; p=0.04) were independently associated with higher odds of having a suppressed viral load. However, being referred for adherence (aOR=0.61, 95% CI: 0.37 - 0.99; p=0.05) and having a previous history of ART exposure (aOR=0.29, 95% CI 0.10 - 0.77; p=0.01) were independently associated with lower odds of having a suppressed viral load.

**Conclusion:**

a higher suppression rate among female patients, suggests that more targeted interventions should be facilitated among HIV-positive male patients linkage to care and initiated on ART. Exposing the patient to a few enhanced support programs hinders progress toward reducing nonadherence, lack of awareness, and inadequate knowledge about the benefits of achieving viral suppression among people living with HIV.

## Introduction

The global Human Immunodeficiency Virus (HIV) epidemic continues to pose a significant public health challenge, with an estimated 38.4 million new HIV infections in 2021, of which 1.5 million were newly diagnosed cases [1]. Most HIV transmissions occur through sexual contact, and despite efforts to curb this phenomenon, only 75% of those aware of their HIV-positive status accessed antiretroviral treatment (ART) [1,2]. South Africa is one of the hardest-hit countries, with an estimated 7.5 million people living with HIV (PLWHIV), constituting 18.3% of the adult population, and 5.5 million of them receiving ART [3]. Sub-Saharan Africa, transitioned from using CD4 count to viral load testing to assess HIV disease progression, aiming to prevent treatment delays and drug resistance [4]. Viral load monitoring defines the amount of HIV viral particles in blood per milliliter which is a critical tool in evaluating ART effectiveness [5]. A primary treatment goal is to achieve Viral Load Suppression (VLS), where viral replication is reduced to undetectable levels (below 400 copies/ml) [6,7]. Viral suppression yields numerous benefits, including reduced transmission risk, improved health outcomes, lower mortality, and a boosted immune system [8,9]. As a result, viral load monitoring is pivotal in assessing ART success and enhancing the quality of life for PLWHIV [10]. Viral load monitoring can be conducted through fresh plasma specimens with nucleic acid quantification or dried blood spots on filter paper cards, the latter being more cost-effective and suitable for resource-constrained settings [11]. Successful viral suppression is typically defined as maintaining a viral load below 400 RNA copies/ml of blood plasma, while virological failure is marked by a viral load exceeding 1000 copies/ml [2,12].

Factors influencing VLS include sociodemographic such as age, sex, marital status, employment, settlement type, social support, and clinical characteristics including WHO staging, pre-ART counseling, adherence, and TB history all play roles [13,14]. While same-day ART initiation and differentiated care models have contributed to improving viral suppression rates [15], non-adherence, lack of awareness, and inadequate knowledge about the benefits of viral load suppression hinder progress [16]. South Africa has thus adopted a universal test and treat strategy, aligning with the 90-90-90 UNAIDS targets, focusing on testing, initiating treatment, and achieving viral suppression [17,18]. Efforts also include youth-friendly services, ward-based primary health outreach teams, telephonic tracing, and e-laboratory tracking systems to enhance patient support [18]. Despite progress, challenges remain in achieving viral load suppression, particularly in sub-Saharan Africa. Countries like Zimbabwe and Mozambique face barriers due to limited resources, resulting in differing levels of viral suppression [3,19]. In South Africa, regional variations in viral suppression rates have also been observed, with some provinces reporting lower rates than others [20]. While South Africa has made significant strides in reducing new HIV infections, viral suppression rates remain varied. It is against this backdrop that this preliminary study addresses the persisting challenge of achieving VLS among PLWHIV, despite implementing strategies like same-day ART initiation and differentiated care. By comprehensively understanding the factors contributing to VLS, the research seeks to inform interventions that significantly enhance retention and adherence to treatment, ultimately improving health outcomes and substantially reducing HIV transmission. Therefore, this study aimed to determine the VLS and factors contributing to VLS among adult patients initiated on a first-line ART regimen in the South sub-district, City of Ekurhuleni.

## Methods

**Study design:** this study employed a retrospective record review approach to analyse the files of an adult patient cohort who commenced first-line ART between 1 April and 30 June 2018. The retrospective record review method was chosen as it leverages pre-existing patient data to address research inquiries centred on patient-centric data [21]. Standardised researcher-administered data collection tools were used to extract information from patient records within the selected health care facilities where ART initiation occurred.

**Study setting:** the study was conducted within the Ekurhuleni South sub-district of the Gauteng province, this study unfolded in an area east of Johannesburg with an expanse of 1.975 km^2^ and a population exceeding four million [22]. In this region, English, Isizulu, Sepedi, Xitsonga, and IsiXhosa are the most widely spoken languages. Among the three sub-districts within the City of Ekurhuleni municipality, the southern sub-district where this study took place stood out. Comprising 32 primary health care facilities and three community health centres, this sub-district provides ART services guided by the National Department of Health (NDoH) and the World Health Organization guidelines [12,23]. Services, in adherence to these guidelines, are rendered free of charge, facilitated by the Three Interlinked HIV Electronic Register (TIER.Net), which aids in electronic medical record creation [24,25].

### Study population

**Target population:** the study involved medical records of adults who initiated first-line antiretroviral regimens from April to June 2018, selected from seven health care facilities in the Ekurhuleni South sub-district. These facilities were chosen from a total of 35, ensuring equal representation of PLWHIV and a range of patient volumes. The selection criteria were based on the number of patients initiated on ART and included seven primary health care facilities. The study´s timeline allowed for an evaluation of viral load suppression during the initial six months and at twelve months post-ART initiation.

**Inclusion and exclusion criteria:** medical records of adult patients (≥18 years) initiated on ART between April and June 2018 within the seven selected Ekurhuleni South sub-district primary health care facilities were included in this study. Excluded from the study were medical records of patients on second and third-line ART regimens, records with incomplete data exceeding 10%, clinical records of patients under 18 years, those re-initiated on ART, and those outside the review cohort (April to June 2018) within Ekurhuleni south sub-district facilities.

**Sample size and sampling technique:** seven health care facilities were conveniently selected based on patient initiation numbers, with 379 patient records sampled, considering a 5% margin of error, 95% confidence interval, and 50% response distribution [26]. Employing convenience sampling, facilities managing over 150 patient ART initiations were selected, and classified by patient headcount. Unique patient numbers were used to randomly select medical records in batches of ten, ensuring equitable representation.

**Data collection:** commenced in selected facilities following ethical approval. It involved the extraction of patient demographic and clinical information using a researcher-designed data extraction tool. Data were collected on variables such as age, gender, employment status, marital status, treatment support, World Health Organization (WHO) staging, same-day initiation, adherence counseling, and more. Data collection took place on selected afternoons and Saturdays, accommodating facility routines. Using TIER Net data previously known as the HIV eRegister [24,25] medical records were filtered and extracted using a structured process. A pilot study was also conducted to ensure feasibility, and the variables used will assist in answering the study objectives. Data were collected by the researcher in designated workspaces within the facilities. Data extracted from medical records were captured in an Excel data extraction tool, ensuring accuracy and completeness. The sample was selected in batches, and any records with incomplete data were replaced. Patient anonymity was upheld by employing unique research codes as identifiers instead of using patients´ names in the data extraction tool.

**Variables:** viral suppression was defined as reducing the function and replication of a virus in the system to as lower up to the set standard of below 400 viral copies per milliliter of blood. Variables used to analyze the characteristics associated with viral suppression were the demographic and clinical variables. Demographic variables included gender dichotomized into (female and male), marital status explained as being (single, married, and divorced), and age defined as being among categories of (18-29,30-39, 40-49, and above 50 years). Occupation is defined as whether the patient is employed, unemployed, or a student. Type of settlement is defined as whether a person is staying in an informal settlement with no basic services or at well-designed formal settlement. Social assistance by family especially by partner during the treatment journey. Clinical variables included Advanced WHO staging defined as a stage where a known HIV-positive patient appears asymptomatic (stage 1-2) and shows symptoms of ill health (stage 3-4). Pre-ART counseling is defined as offering a known HIV patient counseling before starting on ART, Same day initiations are defined as newly tested positive clients being linked to care and started on ART. History of Pulmonary Tuberculosis (PTB) defined previous history of PTB exposure. Pulmonary Tuberculosis treatment completed a defined history of completing the prescribed duration of treatment successfully. Pregnancy status is defined as estimating the number of pregnant patients initiated on ART on first booking, previous history of ART exposure was defined as HIV patient if did take ART before, defaulted, or if taking ART for the first time. Referred for adherence counseling defined the patients who were referred for adhered counseling sessions prior to ART initiation. Six- and twelve-month viral completion and suppression were defined as taking blood for viral suppression at 6 months and 12 months and assessing if the undetectable stage of below 400 milliliters of blood was reached in both intervals of 6 months and at 12 months of testing.

**Statistical analysis:** data were captured, cleaned, coded, and edited in Microsoft Excel 2007. Data analyses were performed using Stata version 16 (StataCorp., College Station, TX, USA). Frequency tabulations and percentages were used to describe categorical variables, whereas means and standard deviations were used to describe continuous variables after testing the data for normality, and non-parametric alternatives were used where applicable. A description of viral suppression prevalence was determined using the proportion of patient records with viral loads below 400 copies/ml over the study population and presented using a bar chart. Univariable logistic regression analysis was performed to determine potential sociodemographic and clinical characteristics associated with viral load suppression. A multivariable logistic regression model was used to determine independent factors associated with viral load suppression among HIV-positive participants, and backward and forward variable selection procedures were followed. The goodness-of-fit and link tests were used to determine the fitness of the multivariable logistic model. P-values below 0.05 (p<0.05) were considered statistically significant.

**Bias:** it was noted that conducting a record review study was prone to information bias. In consideration of the fact that information bias could have led to the variables being under- or over-reported during data collection. Similarly, missing data may have led to hidden or non-response bias. To reduce information bias that results from missing data in the patient files or records, the researcher over-sampled the number of patient records. Again, to minimize bias further the researcher ensured that all the common concerns associated with missing data like incomplete data variables prior to the review were noted.

**Ethical considerations:** ethical approval was obtained from the Sefako Makgatho Health Sciences University Research and Ethics Committee (SMUREC). This study was approved in August 2021 with the ethics number - SMUREC/H/51/2021: PG. Permission was also granted by the Ekurhuleni Health District Research Committee (EHDRC) with the following numbers - NHRD No: GP 202108 098 and Research Project No: 05/11/2021-04.3. Permission to conduct the study was sought from the operational managers of the seven identified facilities in the City of Ekurhuleni southern sub-district.

## Results

**Socio-demographic characteristics from the patient records:** a total of 379 patient records were extracted for this study. Descriptive statistics for participants (Table 1) show that 63.8% were females, and the mean age for the study population was 38.9 years. The majority of the study participants were single (74.7%), unemployed (56.2%), lived in formal settlements (82.9%), and had an average of three dependents. Other trends in patient’s sociodemographic characteristics are displayed (Table 1).

**Table 1:** description of the sociodemographic characteristics by the viral load suppression category

Characteristics		Total	Suppressed	Not suppressed	P-value
		(379; 100.0%)	(285; 75.2%)	(94; 28.8%)	
**Sociodemographic characteristics**					
**Age**	(Mean; DS)	38.9 (10.1)	38.6 (10.2)	39.3 (9.6)	0.60
**Gender**	Male	137 (36.2)	97 (70.8)	40 (29.2)	0.14
	Female	242 (63.8)	188 (77.7)	54 (22.3)	
**Employment status**	Unemployed	213 (56.2)	161 (75.6)	52 (24.4)	0.84
	Employed	143 (37.7)	106 (74.1)	37 (25.9)	
	Grant	13 (3.4)	11 (84.6)	2 (15.4)	
	Student	9 (2.4)	6 (66.7)	3 (33.3)	
	Pension	1 (0.2)	1 (100.0)	0 (0.0)	
**Marital status**	Divorced	2 (0.5)	2 (100.0)	0 (0.0)	0.56
	Single	283 (74.7)	215 (76.0)	68 (24.0)	
	Married	94 (24.8)	68 (72.3)	26 (27.7)	
**Number of dependents**	(Mean; SD)	2.5 (1.7)	2.5 (1.7)	2.6 (1.6)	0.85
**Settlement type**	Informal	65 (17.1)	48 (73.8)	17 (26.2)	0.78
	Formal	314 (82.9)	237 (75.5)	77 (24.5)	
Social support*	No	237 (63.5)	181 (76.4)	56 (23.6)	0.45
	Yes	142 (37.5)	104 (73.2)	38 (26.8)	

* Social support referred to the social assistance received from partner regarding the participant’s HIV status

**Prevalence of viral load suppression:** the prevalence of viral load suppression at 12 months was 75.2% (285/379). By gender, females had a higher prevalence of viral load suppression at 77.7% compared to males at 70.8%; the difference in prevalence among males and females was not statistically significant (p=0.14). Additionally, participants residing in a formal settlement had a slightly higher prevalence of viral load suppression (75.5%) compared to those residing in a formal settlement (73.8%) (p=0.78). There was no statistically significant difference in the prevalence of viral suppression by age (p=0.60). More results on the prevalence of viral load suppression by sociodemographic characteristics are detailed in Table 1.

**Description of the clinical characteristics by the viral suppression category:** clinically, most participants were diagnosed at WHO stages 1 to 2 (93.7%) and received pre-ART counseling (73.1%) with same-day initiation of ART (56.7%). A higher proportion of participants had no previous exposure to ART (76.8%) as well as no history of tuberculosis (92.4%). While on ART treatment, the majority completed the six-month viral load assessment (84.2%), and 71.5% had viral load suppression during the six-month assessment. Further clinical characteristics are displayed (Table 2).

**Table 2:** description of the clinical characteristics by the viral load suppression category

Characteristics			Total		Suppressed		Not suppressed		P-value
			(379; 100.0%)		(285; 75.2%)		(94; 28.8%)		
**Clinical characteristics**									
**Same-day initiation**	No		164 (43.3)		127 (77.4)		37 (22.6)		0.48
	Yes		215 (56.7)		158 (73.5)		57 (26.5)		
**WHO staging**	Stage 1 - 2		355 (93.7)		267 (75.2)		88 (24.8)		0.98
	Stage 3 - 4		24 (6.3)		18 (75.0)		6 (25.0)		
**Pre-ART counseling**	No		102 (26.9)		78 (76.5)		24 (23.5)		0.73
	Yes		277 (73.1)		207 (74.7)		70 (25.3)		
**Previous ART exposure**	No		291 (76.8)		225 (77.3)		66 (22.7)		0.08
	Yes		88 (23.2)		60 (68.2)		28 (31.8)		
**History of TB**	No		350 (92.4)		266 (76.0)		84 (24.0)		0.21
	Yes		29 (7.6)		19 (65.5)		10 (35.5)		
**6 Months viral load completion**	No		60 (15.8)		39 (65.0)		21 (35.0)		0.05
	Yes		319 (84.2)		246 (77.1)		73 (22.9)		
**6 Months viral load suppression**	Not suppressed		108 (28.5)		58 (53.7)		50 (46.3)		<0.00
	Suppressed		271 (71.5)		227 (83.8)		44 (16.2)		
**Referred for adherence**	No		259 (68.3)		205 (79.2)		54 (20.8)		<0.00
	Yes		120 (31.6)		80 (66.7)		40 (33.3)		
ART: antiretroviral therapy, TB: Tuberculosis, WHO: World Health Organization									
Yes		0.45 (0.23 - 0.89)		0.02		0.29 (0.10 - 0.77)		0.01**	
No		Ref				Ref			

ART: antiretroviral therapy, TB: Tuberculosis, WHO: World Health Organization

**Factors associated with viral load suppression among HIV-positive study patients based on the extracted data:** in a multivariable logistic regression model, being female (aOR=7.29, 95% CI: 1.02 - 52.26; p=0.04) and having completed a six-month viral load assessment (aOR=1.85, 95% CI: 1.00 - 3.41; p=0.04) were independently associated with higher odds of having a suppressed viral load. However, being referred for adherence (aOR=0.61, 95% CI: 0.37 - 0.99; p=0.05) and having a previous history of ART exposure (aOR=0.29, 95% CI; 0.10 - 0.70; p=0.01) were independently associated with lower odds of having a suppressed viral load (Table 3).

**Table 3:** univariable and multivariable analysis for factors associated with viral load suppression in HIV-positive patients

Variable	Univariable		Multivariable*	
	cOR (95% CI)	P-value	aOR (95% CI)	P-value
**Sociodemographic characteristics**				
**Age**	0.99 (0.96 - 1.02)	0.42	-	-
**Employments status**				
Unemployed	2.20 (0.43 - 11.29)	0.34	-	-
Employed	2.21 (0.42 - 11.73)	0.35	-	-
Grant	2.34 (0.23 - 23.34)	0.46	-	-
Student	Ref			
**Marital status**				
Single	1.17 (0.60 - 2.26)	0.65	-	-
Married	Ref			
**Number of dependents**	0.95 (0.80 - 1.13)	0.6	-	-
**Gender**				
Female	1.54 (0.83 - 2.87)		7.29 (1.02 - 52.26)	0.04**
Male	Ref		Ref	
**Settlement type**				
Formal	0.84 (0.42 - 1.68)	0.61	-	-
Informal	Ref			
**Social support**				
Yes	0.87 (0.48 - 1.58)	0.65	-	-
No	Ref			
	**Clinical characteristics**			
**Same-day initiation**				
Yes	0.80 (0.47 - 1.37)	0.41	-	-
No	Ref			
**WHO stage**				
Stage 3 - 4	1.55 (0.42 - 5.68)	0.51	-	-
Stage 1 - 2	Ref			
**Pre-ART counseling**				
Yes	0.83 (0.46 - 1.54)	0.57		
No	Ref		-	-
**History of TB**				
Yes	0.50 (0.17 - 1.48)	0.21	-	-
No	Ref			
**6 months viral load completion**				
Yes	5.41 (2.52 - 11.58)	<0.00**	1.85 (1.00 - 3.41)	0.04**
No	Ref		Ref	
**6 months viral load suppression**				
Yes	7.01 (3.83 - 12.83)	<0.00**	-	-
No	Ref			
**Referred for adherence**				
Yes	0.72 (0.41 - 1.28)	0.26	0.61 (0.37 - 0.99)	0.05**
No	Ref		Ref	
**Previous history of ART exposure**				

## Discussion

This study provides valuable insights into the prevalence of viral load suppression and associated factors among HIV-positive study participants. The observed prevalence of viral load suppression at 12 months was 75.2%, indicating a significant proportion of participants achieved successful viral load suppression. It is crucial to note that once an individual starts taking HIV medication as prescribed, they will very likely achieve an undetectable viral load within a period of one to six months [27]. However, variations in viral load suppression prevalence were noted across demographic factors. Contrary to previous studies reporting higher incomplete adherence rates among women [28,29], gender was not significantly associated with viral load suppression in this cohort. This discrepancy might stem from the multivariable logistic regression model´s inclusion of confounding factors affecting the gender-viral load suppression relationship. Interestingly, completing a six-month viral load assessment and being female were independently linked to higher odds of viral load suppression, while referral for adherence and prior ART exposure was associated with lower odds. These findings underscore the importance of adherence monitoring, support, and interventions for referred individuals. Regular assessments remain encouraging, hinting at improved viral load suppression outcomes through consistent monitoring and follow-up [30].

Age did not significantly impact viral load suppression prevalence in this cohort. However, since this study only examined up to 12 months of prevalence, further research is needed to explore age´s potential impact on viral load suppression over extended periods. This complexity highlights viral load suppression´s multifaceted nature, influenced by various factors. Furthermore, the association between referral for adherence and reduced viral load suppression underscores the necessity of offering comprehensive support for struggling individuals, including counseling, reminders, and adherence-focused interventions [31]. In this study, viral load suppression prevalence was 75.2% at 12 months. Factors tied to increased suppression odds included being female and completing a six-month viral load assessment, while adherence referral and prior ART exposure correlated with decreased odds. The study results emphasize the need for immediate linking of newly initiated HIV-positive patients to Ward Based Outreach Teams as well as interventions targeting adherence and support for those with an ART exposure history. The study´s findings show that certain factors do influence viral load suppression among HIV-positive individuals. Further research should investigate these factors and potential interventions to improve medication adherence and enhance viral load suppression outcomes. While this study did not adjust for adherence, a recognized limitation, previous research established adherence´s pivotal role in achieving viral load suppression [32].

These findings hold significant implications for HIV treatment and care. Understanding factors influencing viral load suppression informs interventions and strategies to enhance treatment outcomes [33]. Continual viral load monitoring in low and middle-income countries is crucial for assessing viral suppression levels, contributing to global epidemic control [34]. Early viral suppression, as demonstrated by [35], aids in reducing onward HIV transmission, new incidences, and HIV-related health care costs. The generalisability of the study´s findings is constrained by the limited sample size, warranting careful interpretation. It is worth highlighting that record review studies, as observed here, are particularly susceptible to the influence of incomplete data. Notably, even though the electronic data capture sheet encompassed all pertinent variables, the data input by service providers remained constrained, resulting in data limitations. A notable proportion of patient records exhibited data gaps and incompleteness, further underscoring the impact. The study results showed that more females were initiated on ART and achieved viral suppression, and that early initiation and doing 6 months viral measurements put patients at advantage to reach VLS as early as 6 months of ART initiation.

## Conclusion

Based on the research findings, a comprehensive approach is recommended to enhance VLS outcomes and optimize HIV management. The higher prevalence of VLS among women warrants targeted support programs to overcome potential adherence barriers and promote regular viral load assessments. The authors recommend that adherence support programs should be strengthened to encompass various aspects, including immediate linking of newly initiated HIV-positive patients to WBPHCOT, counseling, reminders, and personalized interventions. Special attention should be given to individuals referred for adherence, ensuring comprehensive assistance to improve their suppression outcomes. Encouraging regular viral load monitoring, especially completing a six-month assessment, is crucial for sustained VLS. Continual viral load monitoring facilitates assessments of viral suppression levels, contributing significantly to achieving global epidemic control. The recommendations underscore the need for tailored interventions, comprehensive support, sustained monitoring, and global collaboration to optimize VLS outcomes, reduce HIV transmission, and enhance overall HIV treatment and care.

### What is known about this topic

- That being diagnosed with HIV-positive is no more a death sentence or life-threatening disease but considered a chronic controlled disease;
- It has been evidenced that every HIV- positive patient started on antiretroviral treatment and with good compliance can achieve viral suppression within the first six months of initiation;
- The more people on ART achieve viral suppression and contribute towards reduced spread of new HIV infection.

### What this study adds

- Completing six-month viral load assessment and being a female in this study were independently linked to a higher odd of viral suppression;
- Patients referred for adherence support and those with history of prior antiretroviral exposure were correlated with decreased odds;
- Targeted interventions should be facilitated to improve immediate linkage to treatment, adherence monitoring and support for referred individuals and those with prior exposure to antiretrovirals.
